# Evaluation of transperineal ultrasound imaging as a potential solution for target tracking during hypofractionated radiotherapy for prostate cancer

**DOI:** 10.1186/s13014-018-1097-8

**Published:** 2018-08-20

**Authors:** Bin Han, Mohammad Najafi, David T. Cooper, Martin Lachaine, Rie von Eyben, Steven Hancock, Dimitre Hristov

**Affiliations:** 1 Department of Radiation Oncology, School of Medicine, Stanford University, 875 Blake Wilbur Drive, Room G203, Palo Alto, CA 94304 USA; 2 Elekta Ltd, 2050 Bleury Suite 200, Montréal, QC H3A 2J5 Canada

**Keywords:** Prostate cancer, Ultrasound, Tracking, Prostate motion, Radiotherapy, Image-guidance

## Abstract

**Background:**

Emerging hypofractionated prostate radiotherapy regimens require solutions for accurate target tracking during beam delivery. The goal of this study is to evaluate the performance of the Clarity ultrasound monitoring system for prostate motion tracking.

**Methods:**

Five prostate patients underwent continuous perineum ultrasound imaging during their daily treatments. Initial absolute 3D positions of fiducials implanted in the prostate were estimated from the KV images. Fiducial positions in MV images acquired during beam delivery were compared with predicted positions based on Clarity 3D tracking. The uncertainty in the comparison results was evaluated in a phantom validation study.

**Results:**

Continuous real-time ultrasound motion tracking was recorded in 5 patients and 167 fractions for overall of 39.7 h. Phantom validation of the proposed procedure demonstrated that predicted and observed fiducial positions agree within 1.1 mm. In patients agreement between predicted and actual fiducial positions varied between 1.3 mm and 3.3 mm. On average ultrasound tracking reduced the maximum localization error in patients by 20% on average. With the motion corrected, the duration prostate beyond 1 mm from its initial treatment position can be reduced from 37 to 22% of the total treatment time.

**Conclusion:**

Real-time ultrasound tracking reduces uncertainty in prostate position due to intra-fractional motion.

**Trial registration:**

IRB Protocol #27372. Date of registration of trial: 12/17/2013.

## Background

Hypofractionated prostate treatment is contingent on having the technical means to deliver treatments with minimum risk of mis-targeting. Various type of fiducials are usually implanted and tracked via x-ray images or electromagnetic signals, but this method is invasive and the fiducials could migrate over the time [1]. Using kV on board imaging results in extra imaging dose to patients and may increase the secondary cancer risks [2]. Electromagnetic tracking of implanted transponders is being utilized clinically, but transponder-caused artifact in magnetic resonance (MR) imaging affects the disease management process from planning to follow-up. Therefore, hypofractionated prostate treatment’s wide adoption is contingent on providing solutions for accurate tracking during beam delivery when accurate targeting is most critical.

Ultrasound, as a non-invasive, real-time and inexpensive modality for imaging soft tissue, can be a powerful tool for guidance of radiation treatment and minimizing the risk of mis-targeting. It is possible to use the imaging modality for both simulation and treatment with no additional imaging dose [3]. Recently a transperineal imaging system (Clarity®, Elekta, Stockholm, Sweden) was introduced to acquire volumetric ultrasound images for either pre-treatment target localization (daily Image-guided radiation therapy (IGRT)) or real time intra-fractional prostate tracking. Several groups have reported their initial clinical experience for these applications [4–9]. In patients, the accuracy of the system has been investigated only for its IGRT application [4, 7, 9], but not for intra-fractional tracking. Given that Clarity system uses different workflow, acquisition, and image processing algorithms for intra-fractional tracking, the accuracy of its performance for this application remains unknown as it cannot be inferred from data reported for the IGRT application.

We have previously evaluated the Clarity prostate tracking performance in phantoms [10] and we have found that the system was able to track a target in a phantom with an average error of ~ 0.5 mm. However, this error should be interpreted as the best achievable accuracy and it is likely not representative of system tracking performance in real treatment scenarios. Thus, the goal of this work is to evaluate the system tracking performance in patients under treatment. To this end we systematically compare and correlate the Clarity estimated prostate position to the prostate position visualized by “pseudo-cine” on-treatment MV images of fiducials implanted in the prostate. We quantify sources of uncertainty in this comparative evaluation and discuss the clinical utility of the system in view of our findings.

## Methods

### Treatment procedure and data acquisition

For this study, with IRB approval (Institutional Review Board) transperineal ultrasound imaging of the prostate was performed with the Clarity® system (Elekta, Stockholm, Sweden) for five prostate patients during simulation and treatment delivery. Briefly the process was as follows.

At simulation, a reference 3D ultrasound image was acquired and fused with the planning computed tomography (CT) using the Automated Fusion and Contouring Workstation (Elekta, Stockholm, Sweden). The CT contours of several structures (prostate, bladder, and rectum) were transferred to the reference ultrasound image. The prostate contours were set as an image guidance volume with minor adaptions to better represent edges in the ultrasound images. Once approved, the treatment plan was imported in the Clarity system to localize the treatment isocenter position within the reference ultrasound. Before treatment delivery, a 3D guidance ultrasound image was acquired and the prostate was manually identified based on the predefined reference ultrasound images and the image guidance volume. A 3D shift vector was then calculated by the Clarity® system so that the spatial relation between the treatment isocenter and the prostate center reflected in the guidance ultrasound image matched the intended spatial relation captured in the reference ultrasound image. After applied the 3D shift vector, the Clarity system was then set in a tracking mode to monitor all subsequent 3D displacements of the prostate with respect to the reference prostate position as captured in the guidance ultrasound image.

Next, a pair of orthogonal kV images was acquired with an On-Board Imager® (OBI) on a Varian 23EX Linac. Registration of this kV pair to a corresponding pair of DRRs (Digitally Reconstructed Radiographs) was performed based on 4 fiducials previously implanted in the prostate. The prostate was then repositioned and a volumetric modulated arc therapy (VMAT) treatment initiated. Exit MV portal images were continuously acquired during the VMAT delivery in a cine or pseudo-cine mode.

**Fig. 1:**
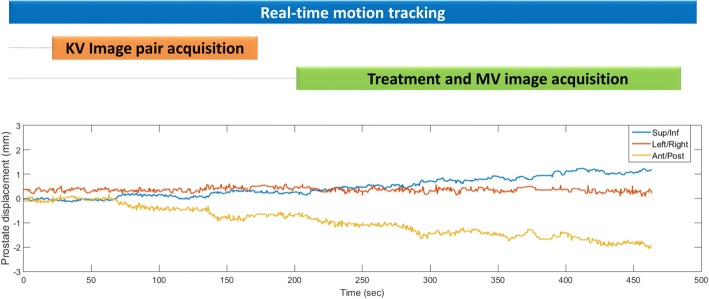
A representative example of a patient’s prostate displacements throughout a treatment session. Note that KV image pair acquisition also includes image registration and couch repositioning

With this process real-time prostate tracking was performed continuously throughout standard treatment sessions including x-ray imaging and delivery (Fig. 1). Since reflective marker arrays are affixed to the top of the transperineal probe and the treatment couch, the Clarity system can track independently both prostate and couch motions. This allowed couch motion introduced after kV-DRR matching to be identified and accounted for in the data analysis.

Normal assumption with x-ray based image guidance is that no motion occurs during kV pair image acquisition, CBCT scanning and radiation delivery. The add-on ultrasound treatment monitoring allowed analysis of targeting errors that would arise from target motion with standard x-ray based image guidance. These are reported in In-vivo evaluation of transperineal ultrasound tracking section.

### Image analysis and data processing

In-vivo evaluation of the Clarity 3D tracking accuracy requires that the 3D prostate trajectory be known as ground truth. While pairs of orthogonal MV images can be used to triangulate the positions of the implanted fiducials during treatment, this approach assumes that the prostate remains stationary during the acquisition of the MV pair, an assumption which is not valid (Fig. 1). Given that only the 2D positions of (some) fiducial markers in the MV images are available as ground truth during treatment, we compare these measured 2D positions to expected positions simulated from the initial locations of the fiducials based on kV-kV imaging and the prostate trajectory recorded by Clarity. Mathematically, if *P*(*x*_0_, *y*_0_, *z*_0_) is the initial in-room position of a fiducial determined by kV-kV stereo triangulation, the fiducial projection *p*^*t*^(*u*, *v*) onto an MV portal image acquired at a gantry angle *θ* and time *t* can be determined as:

1 $$ {p}^t\left(u,v\right)={\mathrm{T}}_{{\mathrm{P}}_{\mathrm{r}}}\left(\theta, \kern0.5em SAD,\kern0.5em SID\kern0.5em {k}_u,\kern0.5em {k}_v,\kern0.5em {u}_0\kern0.5em {v}_0\right){T}_0^tP\left({x}_0,\kern0.5em {y}_0,\kern0.5em {z}_0\right) $$

In this expression ${T}_0^t$ is the Clarity reported tracking transformation such that the position of the fiducial at time *t* is $P\left({x}_t,{y}_t,{z}_t\right)={T}_0^tP\left({x}_0,{y}_0,{z}_0\right)$ and ${\mathrm{T}}_{{\mathrm{P}}_{\mathrm{r}}}\left(\theta, \kern0.5em SAD,\kern0.5em SID,\kern0.5em {k}_u,\kern0.5em {k}_v,\kern0.5em {u}_0,{v}_0\right)$ is a transformation that projects the point *P*(*x*_*t*_, *y*_*t*_, *z*_*t*_) onto a point *p*^*t*^(*u*, *v*) within an MV portal image. ${\mathrm{T}}_{{\mathrm{P}}_{\mathrm{r}}}\left(\theta, \kern0.5em SAD,\kern0.5em SID,\kern0.5em {k}_u,\kern0.5em {k}_v,\kern0.5em {u}_0,\kern0.5em {v}_0\right)$ is determined by the gantry angle *θ*, source-to-axis distance (SAD), source-to-imager distance (SID), pixel size (*k*_*u*_, *k*_*v*_), portal imager coordinate system offset (*u*_0_, *v*_0_), and additional system corrections obtained from the IsoCal geometric calibration system [11] to correct for imaging offsets and gantry sag. Given that the actual fiducial position ${p}_m^t\left(u,v\right)$ in the portal image is known, the norm of the difference vector ${p}^t\left(u,v\right)-{p}_m^t\left(u,v\right)$ when scaled back from the imaging plane to a parallel plane going through the isocenter provides a measure for the error in the tracking transformation ${T}_0^t$.

### Phantom validation

**Fig. 2:**
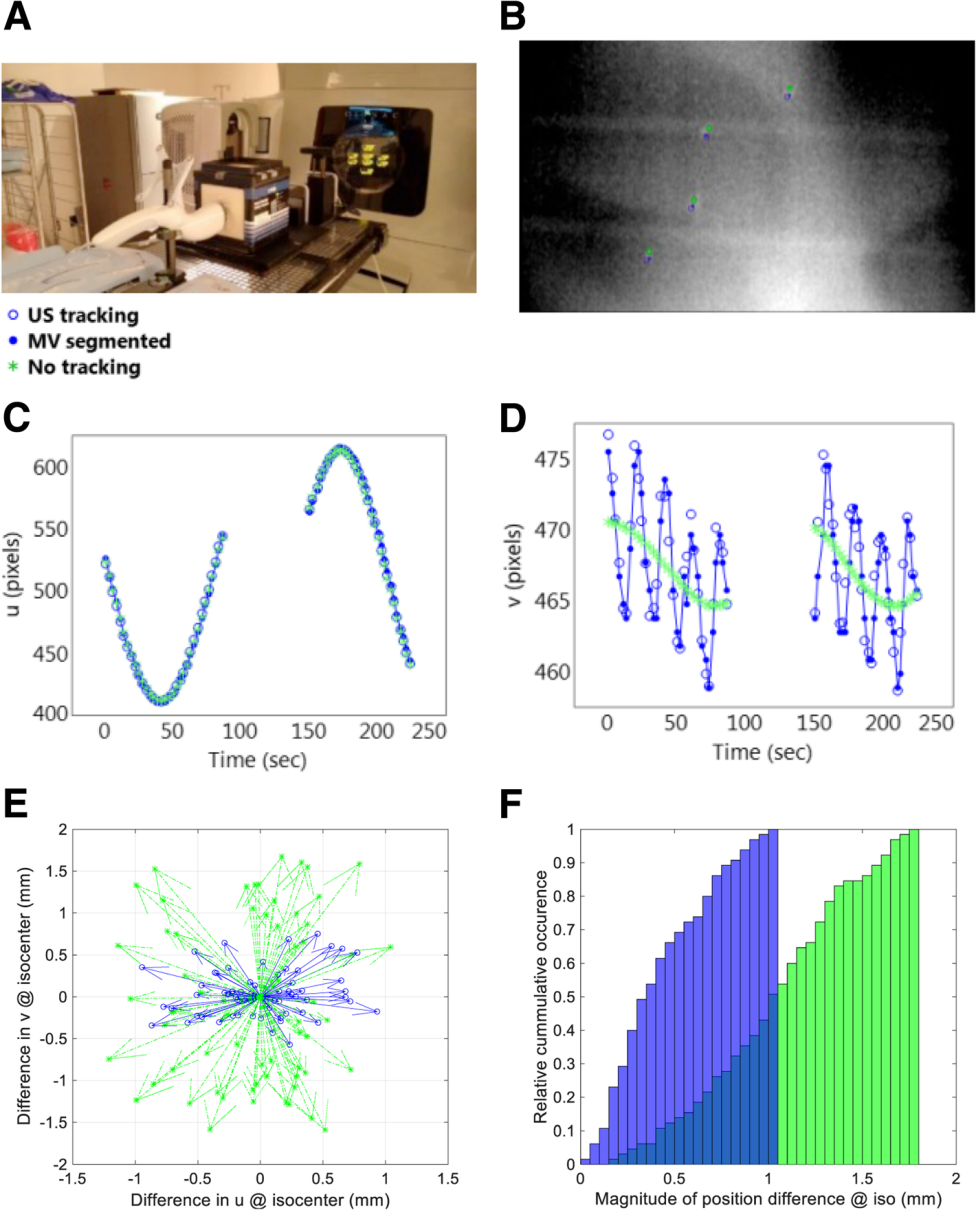
**a** Experimental setup for phantom validation. **b** A sample MV image with indicated positions of the segmented fiducials (solid blue circles), predicted positions of the fiducials based on ultrasound tracking (hollow blue circles), and predicted positions of the fiducials assuming no motion (green asterisks). **c** Pixel coordinate (u) of fiducial 1 at different times during the MV arc beam delivery. **d** Pixel coordinate (v) of fiducial 1 at different times during the MV arc beam delivery. **e** Difference vectors (scaled at isocenter) between mean predicted and mean actual (MV segmented) positions of the 4 fiducials. **f** Relative cumulative distribution of the magnitudes of the difference vectors shown in (**e**)

The proposed procedure was first validated in a phantom study. A multi-modality pelvic phantom (CIRS, Norfolk, VA) with four fiducials implanted in “prostate” was placed on a motion platform (CIRS, Norfolk, Virginia) in contact with the transperineal ultrasound transducer (Fig. 2a). After 3D localization of the fiducials with orthogonal kV imaging, phantom motion with a sawtooth pattern was initiated in longitudinal direction. The programmed peak-to-peak motion amplitude was limited to 4 mm to prevent decoupling of the ultrasound transducer from the pelvic phantom. MV images were subsequently acquired during arc delivery. The accuracy and precision of the ultrasound tracking was subsequently evaluated with the image analysis described in Background section above.

## Results

### Phantom validation

Figure 2b presents an example of a MV image showing the actual positions of the segmented fiducials, the predicted positions of the fiducials based on ultrasound tracking, and the predicted positions of the fiducials assuming no motion. Figure 2c and d show the segmented and predicted image pixel coordinates of fiducial 1 at different times during the MV arc beam delivery. Figure 2d clearly demonstrates the sawtooth pattern of the fiducial actual pixel position introduced by the phantom motion. Figure 2d shows that incorporating the ultrasound tracking information allows the fiducial predicted position to track the fiducial actual position in contrast to the case where motion is not tracked. Figure 2e illustrates the difference vectors between mean predicted and mean actual positions of the 4 fiducials. The mean differences between actual and tracked fiducial positions were [0.0 ± 0.4 0.0 ± 0.3]mm. Figure 2f demonstrates that the absolute agreement between actual and predicted fiducial positions was within 1.1 mm in this controlled phantom experiment. Thus, we conclude that the precision of our procedure for evaluating the Clarity performance against fiducial markers is 1.1 mm or better.

### Prostate motion summary

The patients enrolled in this study underwent 195 treatment fractions including 70 boost fractions. The ultrasound monitoring was available for 167 fractions. MV fiducial tracking and ultrasound monitoring were both available for 39 boost fractions. Continuous ultrasound motion tracking was done for 39.7 h.

**Fig. 3:**
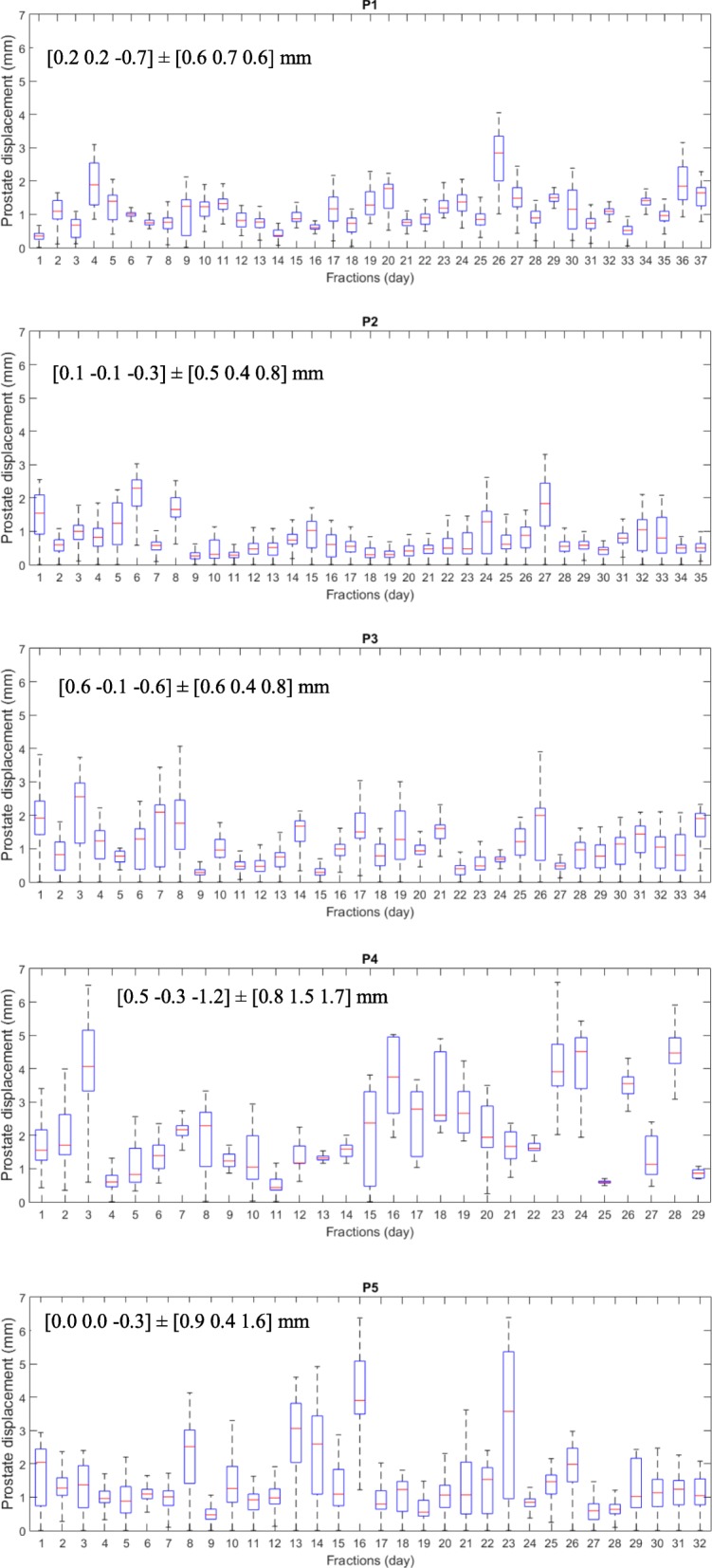
RMS of the prostate displacements in each fraction for all five patients. Mean prostate displacements and standard deviations along IS (Inferior-Superior), LR (Left-Right) and AP (Anterior-Superior) directions are also given

Figure 3 illustrates the RMS (root mean square) of the prostate displacements in each fraction for all patients indicating the variability of prostate motion across patients and fractions.

The ultrasound measured mean prostate displacements across all patients and fractions were 0.3 ± 0.7 mm, − 0.1 ± 0.7 mm, and − 0.6 ± 1.1 mm in IS (Inferior-Superior), LR (Left-Right) and AP (Anterior-Superior) directions. The RMS was 0.7 ± 1.5 mm.

The average motion of the prostate during kV image acquisition was 0.3 mm with a maximum motion of 0.8 mm. The average motion of the prostate between kV/CBCT acquisition and treatment delivery was 0.6 mm with a maximum motion of 1.3 mm over time periods ranging from 3.7 to 8.6 min.

### In-vivo evaluation of transperineal ultrasound tracking

**Fig. 4:**
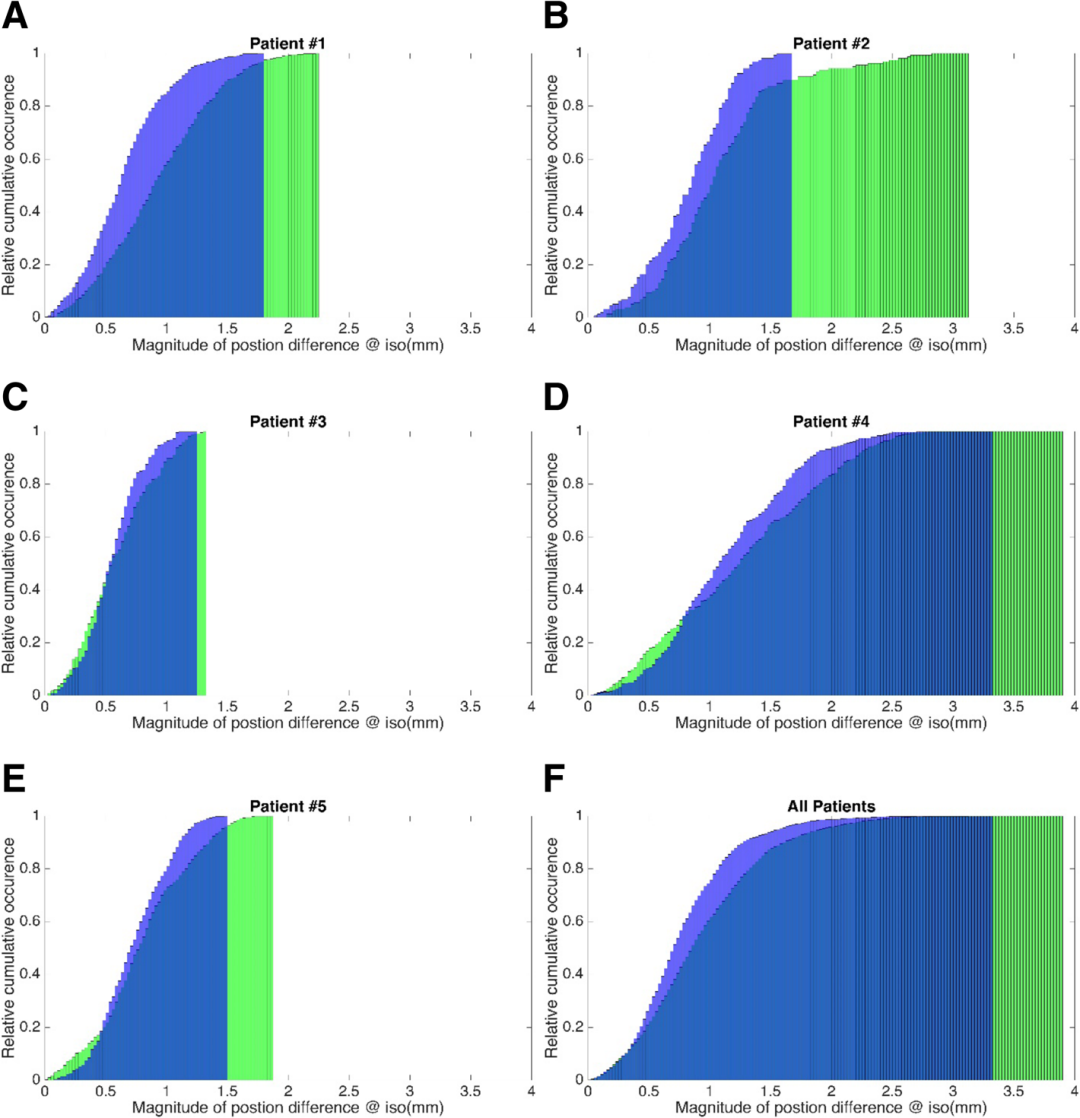
**a**-**e** Relative cumulative distributions of the magnitudes of the difference vectors (scaled at isocenter) between mean predicted and mean actual (MV segmented) fiducial positions for individual patients. Predicted positions are calculated with (blue) and without (green) ultrasound tracking. **f** Same as in A-E with aggregate data for all patients

For individual patients and for all patients, Fig. 4 illustrates the cumulative distributions of the magnitude of difference vectors (scaled at isocenter) between mean predicted and mean actual (MV segmented) positions of the fiducials whereby the predicted positions are calculated with and without ultrasound tracking. For patients 1 to 5, during treatment MV cine and pseudo-cine images were available for 13, 7, 5, 10 and 4 fractions respectively with corresponding ultrasound tracking data recorded for 34.6 min of beam-on time.

Table 1 summarizes some of the data presented in Fig. 4. It illustrates that in comparison to the phantom results presented in Fig. 2f, the maximum differences between actual and predicted ultrasound-tracked fiducial positions in patients exceeded 1.1 mm and varied from 1.3 mm to 3.3 mm depending on the patient. Table 1 further demonstrates that in 95% of the measured instances the differences between actual and predicted ultrasound-tracked fiducial positions were smaller than 1.1 mm to 2.4 mm depending on the patient.

**Table 1:** Summary of the magnitudes of the max and 95% position differences (scaled at isocenter) between predicted and actual (MV segmented) fiducial positions for individual patients

Patient #	Magnitude of position differences @ iso (mm)			
	Maximum		At 95% relative cumulative occurrence	
	Without tracking	With tracking	Without tracking	With tracking
1	2.3	1.8	1.7	1.2
2	3.1	1.7	2.2	1.3
3	1.3	1.3	1.1	1.0
4	3.9	3.3	2.4	2.1
5	1.9	1.5	1.5	1.2

Position differences are calculated with and without ultrasound tracking

## Discussion

The results from this study suggest that transperineal US is feasible for real-time prostate tumor monitoring. In a phantom the maximum tracking error was 1.1 mm whereas in patients the maximum tracking error varied between 1.3 mm and 3.3 mm depending on the patient.

The tracking error is generally small, and one reason may be the presence of prostate motion occurring during the acquisition of the kV image pair (mean 0.3 mm, max 0.8 mm). Such motion during the kV image pair acquisition introduces bias (error) in the initial 3D ground truth position of the fiducials. Additionally, prostate rotations occurring intra-fractionally introduce further discrepancies between Clarity and MV imaging since only translations are reported by the Clarity clinical system. These discrepancies are already captured in the presented in-vivo data and reflect the limitations of the Clarity tracking in the presence of rotations. Another reason is the patient- and operator-dependent variation in image quality, as illustrated by the different performance of the ultrasound tracking in patients 2 and 4 even though these patients similarly exhibit large and frequent prostate movements. Thus with acquisition of optimal ultrasound images, the ultrasound system is feasible for real time prostate monitoring.

Given the observed tracking uncertainties and the relatively small and infrequent prostate displacements, a question arises whether ultrasound tracking performance can in fact help monitor and correct for intra-fractional prostate motion. Table 1 demonstrates that indeed for all patients, ultrasound tracking reduces the localization uncertainty due the prostate motion by 20% on average. This is especially true for patients with larger prostate displacements such as for instance patient 2 for whom the maximum localization error was reduced by 45% (from 3.1 mm to 1.7 mm). Summarizing the data for all patients, Fig. 4f further illustrates that the fraction of beam on-time during which a prostate is displaced by more than 1 mm is reduced from 37% with no tracking to 22% with tracking. Figure 4f also illustrates that with tracking, in 95% of the evaluated instances, the prostate can be localized within 1.6 mm in the beam-eye view.

The use of 2D MV portal imaging for ground truth measurements is a limitation of our study. First, some of the discrepancies observed are caused by the uncertainty in localizing the prostate fiducials within the MV images. Second, evaluation of the ultrasound tracking accuracy is inherently performed in 2D as it is limited to the plane of the MV images. Alternative study design would be to compare ultrasound tracking to accurate in-vivo tracking with electromagnetic (EM) transponders, but the feasibility of such an approach is yet to be established as there may be hardware interferences between the antenna used for EM tracking and the optical camera used for ultrasound tracking.

## Conclusion

Transperineal US is feasible for real-time prostate tumor monitoring and can reduce the intrafractional uncertainty in the prostate position.

## Abbreviations

CT: Computed tomography
DRRs: Digitally Reconstructed Radiographs
IGRT: Daily Image-guided radiation therapy
IRB approval: Institutional Review Board
MR: Magnetic resonance
OBI: On-Board Imager®
VMAT: Volumetric modulated arc therapy
